# Prostate Cancer Knowledge and Attitude Toward Screening Practices Among Men 40 and Over in the Jazan Region, Saudi Arabia

**DOI:** 10.1155/2024/2713372

**Published:** 2024-11-06

**Authors:** Anas Elyas, Mohamed Salih Mahfouz, Abdulkarim Zain Ahmed Suwaydi, Osama Ali Alotayf, Abdullah Omar Tayri, Bandar Fahad Daghriri, Abdulrahman Abdu Daghreeri, Faisal Ahmed Hattan, Mohammed Maqbul Akkam

**Affiliations:** ^1^Department of Family and Community Medicine, Faculty of Medicine, Jazan University, Jazan, Saudi Arabia; ^2^Faculty of Medicine, Jazan University, Jazan, Saudi Arabia

**Keywords:** attitude, cancer, Jazan, knowledge, prostate, prostate cancer, Saudi Arabia, screening

## Abstract

**Background:** Prostate cancer (PCa) is the second most prevalent malignancy among males and ranks as the fifth primary cause of mortality worldwide, underscoring its substantial impact on public health. Notably, there is a lack of research focused on PCa within the context of Saudi Arabia. Consequently, this study endeavours to elucidate the knowledge, attitudes, and screening practices related to PCa among males in Jazan, Saudi Arabia.

**Methodology:** A cross-sectional survey was carried out on males over 40 years old in the Jazan region of Saudi Arabia between December 2022 and March 2023. The survey used a web-based questionnaire containing questions about sociodemographic characteristics, PCa knowledge, and attitudes toward screening practices. Descriptive and inferential statistical analysis evaluated the participants' knowledge and attitudes toward PCa screening.

**Results:** Out of the 468 male participants aged over 40 years, approximately 44% demonstrated limited awareness about PCa. Intriguingly, 60.3% of participants showed a positive attitude toward PCa screening. Moreover, 35.7% reported consulting a urologist for screening purposes. Furthermore, 25.6% had undergone a prostate-specific antigen (PSA) test, with the predominant rationale (25.8%) being medical advice. Multivariate analysis revealed that being married (adjusted odds ratio (AOR) = 4.5, *p* = 0.011) and having a family history of PCa (AOR = 4.6, *p* = 0.001) were significant predictors of heightened PCa awareness. Concurrently, a history of PCa (AOR = 6.8, *p* = 0.001) and holding a postgraduate qualification (AOR = 5.5, *p* = 0.024) emerged as significant determinants of proactive practices toward PCa.

**Conclusion:** The results revealed a significant lack of knowledge regarding PCa and the screening practices associated with it among the study participants. These results emphasize the urgent need to provide men with comprehensive information about the benefits and limitations of PCa screening in order to enable them to make more informed decisions.

## 1. Introduction

Prostate cancer (PCa) is a significant global malignancy and a leading cause of cancer-related deaths in males, ranking as the fourth most common disease worldwide [1, 2]. Additionally, there has been an increase in PCa incidence and mortality rates in Arabic countries, with further rises expected in the future [3, 4]. PCa is one of the most common cancers among men in Saudi Arabia, and the incidence rate has been increasing. A recent retrospective study, based on the Saudi Cancer Registry, covered the period from January 2008 to December 2017 and involved 3607 patients with PCa. The study revealed that the incidence rates of PCa ranged from 0.2 to 1.4 per 100,000 individuals and indicated a high metastasis rate (31.4%) among PCa patients [5].

In recent years, survival rates for PCa have increased due to early detection, improved treatment options, and ongoing care for survivors [6]. Consistent screenings and medical exams are essential for preventing PCa. The digital rectal exam (DRE) and prostate-specific antigen (PSA) tests are commonly used clinical tools. [7]. Moreover, significant research has been focused on DRE and serum PSA evaluations in the context of PCa screening [7–12]. The American Urological Association (AUA) advised males between the ages of 55 and 69 to undergo PSA screening for PCa [13].

Many studies have endeavoured to gauge male cognizance regarding PCa, revealing variegated levels of awareness and engagement in different regions [14–19]. Multiple factors have been suggested to impact the usage of PCa screening. These include the perceived risk of PCa, limited knowledge about the disease, and the various PCa screening methods available [20, 21]. Research conducted in Saudi Arabia revealed varying levels of knowledge and negative attitudes toward PCa, as well as low rates of screening practice. In a study conducted by Alothman et al., among males aged 40 and above, 55.2% of participants had an inadequate level of knowledge about PCa and the PSA test, while 53.1% had a negative attitude [22]. A recent study conducted among men in Medina, Jeddah, and Mecca, Saudi Arabia, found that 77% of the participants had heard about PCa. In comparison, 52.5% were aware of PCa screening tests. Another research conducted in Riyadh in 2021 documented that 64% of males exhibited sufficient knowledge about PCa, while 70% of them believed that screening for PCa is highly important. Around 23% of the participants had undergone either a PSA test or a DRE [23].

Although numerous studies [22–26] have been conducted in various regions of Saudi Arabia to investigate the knowledge, attitude, and practice toward PCa and its screening methods, none have been conducted in Jazan, Southwest Saudi Arabia. Therefore, this study is aimed at assessing the knowledge and attitudes toward PCa screening practices among men aged 40 years and older in Jazan, Saudi Arabia. Additionally, we sought to elucidate the current screening practices for PCa being undertaken in this population.

## 2. Materials and Methods

### 2.1. Study Design, Settings, and Sampling

A descriptive cross-sectional study evaluated PCa awareness, knowledge, and screening practices among men aged 40 and above in the Jazan region. Jazan region is situated in the southwestern sector of the Kingdom of Saudi Arabia, bordered by the Red Sea. As of the 2020 census, this region had a population of 1.6 million, with those aged 40 and above accounting for 265,092, encompassing both Saudi and non-Saudi residents. The sample size was determined to be 478, using a knowledge prevalence of 50%, a 95% confidence interval (CI), a 5% margin of error, and a 25% nonresponse rate. A nonrandom convenience sampling strategy was adopted to recruit the study participants.

### 2.2. Study Participants and Recruitment Process

The target participants were males aged 40 or older living in the Jazan region. The main inclusion criteria were being a male over 40, having racy in Arabic, being willing to participate, and having residency in the Jazan region. To preserve participant confidentiality, identifiable data such as names or ID numbers were not collected. The survey link was distributed to the general population by asking persons who fit our study's inclusion criteria to fill in the questionnaire after consent. Participants were promulgated via social media platforms, including WhatsApp, Telegram, and Twitter. Each participant has one opportunity to participate in the survey, which was prepared via Google Forms.

### 2.3. Study Instrument and Measures

Data was collected using a self-administered online questionnaire with 34 questions. The questionnaire was designed to be simple and clear and divided into four sections. The first section covered sociodemographic details such as age, educational level, family history of PCa, marital status, place of origin, and monthly income. The second section focused on questions about PCa knowledge, while the third section included questions about participants' attitudes toward PCa screening. The fourth section addressed screening practices among the study participants. The outcome variables were computed based on related questions and then categorized. Knowledge scores ranged from 0 to 17, with correct answers receiving one point and incorrect or “I don't know” responses receiving zero points. Scores of ≥ 12 were considered “*good*” knowledge, scores between 8 and 11 were classified as “*fair*” knowledge, and scores of < 8 were labeled as “*poor*” knowledge. Attitude scores were dichotomized into positive attitudes (greater than 40) and negative attitudes (less than 41). Additionally, a pilot study was conducted with 20 participants to test the clarity and comprehensibility of the Arabic translation version of the previously validated English questionnaires. The internal consistency of the questionnaire was calculated to be 0.70 using Cronbach's alpha test.

### 2.4. Data Analysis

Data extraction from the questionnaire was processed and examined using the Statistical Package for the Social Sciences (SPSS) Version 26. Preliminary steps involved inspecting the dataset for any missing values. After this, all variables underwent descriptive analysis. The chi-squared or Fisher's exact test was applied to investigate the association between background characteristics and PCa awareness, knowledge, and screening practices. Logistic regression models were established to pinpoint factors correlating with PCa awareness, knowledge, and screening practices, with odds ratios and their CIs computed. A threshold *p* value of < 0.05 was set as the marker for statistical significance.

### 2.5. Ethical Considerations

Before data procurement, all requisite official authorizations were secured. All potential participants were briefed on the study's objectives and reassured of the study's nonharmful nature, data anonymity, and comprehensive confidentiality. Informed consent was sought from all participants. In January 2022, the Jazan University Research Ethics Committee (REC-44/06/454) granted ethical clearance.

## 3. Results

Four hundred sixty-eight participants (97.9%) of the planned 478 men aged 40 years and above completed the survey. The majority of the participants were Saudis (98.1%). The age of participants was categorized into three groups: adults (40–49 years), (50–59 years), and elderly (above 60 years). Most were between 40 and 49 years old (56.6%). Regarding the educational level, most participants have a college degree or higher (70.9%). About 34.4% of respondents have a monthly income between 10,000 and 15,000 Saudi Riyal (SR). Furthermore, 80.8% of the participants were married, while 66.7% had a negative family history of PCa (Table 1).

The study found that nearly half of the participants (44%) had poor knowledge of PCa, while 36.8% had fair knowledge, and only 19.2% had good knowledge of the disease. Significant associations were found between knowledge and sociodemographic variables such as marital status, educational level, residence, and monthly income, with a *p* value of less than 0.05 for all. However, no significant association was found between knowledge and age group or nationality, with *p* values of 0.078 and 0.965, respectively. This indicates that participants with higher education, married status, and higher monthly income tend to know more about PCa than others (Table 2).

The men between 50 and 59 years old (68.5%) displayed the highest percentage of positive attitudes. Based on marital status, the widower (93.3%) had a high percentage of negative attitudes, while the married men (65.3%) had positive attitudes. The men who were postgraduates (85.3%) had an excellent attitude, while those with only primary education (79.2%) had a negative attitude in a high percentage. Men with a monthly income between 10,000 and 15,000 SR exhibited a higher percentage of positive attitudes (69.6%). Lastly, the association between attitudes toward PCa screening and sociodemographic variables in the table was significant (*p* value < 0.05 for all), except for nationality and residence, with *p* values of 0.692 and 0.254, respectively (Table 3).

Participants had a negative attitude toward the “effectiveness of DRE (anal exam) and its importance” where the percent agreement was less than 40%, in the same context as their “beliefs about the importance and effectiveness of PSA (blood test) as an important diagnostic tool” (50%–75%). Quite a percentage of “the participants aren't sure about doing PCa examination tests as they believe it is expensive” (48.9%). The highest percentage of disagreement attitude statements (58.1%) were “doing PCa examination tests as they increase their anxiety and worries.” Over 60% (61.2%) confirmed that “they are committed to doing the required diagnostic procedures and committed to the physician's advice even if busy,” as shown in Table 4.

The results of participants' practices regarding PCa screening according to age groups showed that most of our participants who did not visit a urologist clinic for a screening practice are between 40 and 49 years old (72.5%). Among participants, the age bracket with the highest recognition of visiting the urologist for screening practices and requesting the PSA test was over 60 (51.1%). Most of the participants age group of who did the PSA test were above 60 years old (31.5%), followed by those between 50 and 59 years old (30.6%), whereas the lowest group who did not do the PSA test were those aged between 40 and 49 years old (78.5%). In addition, the number of participants who will do the test in the future was highest in those aged (50–59) and (40–49) years old (75.7% and 70.2%), respectively. Regarding the association between participants' age groups and their practices of PCa screening, all the statements showed significant association (*p* value < 0.05) except those who did the PSA test, the reason for avoiding the PSA test, and the intention to do the PSA test in the future (*p* value > 0.05 for all) (Table 5).

The multivariate logistic regression analysis was used to explore the factors that could determine participants' practices, knowledge, and attitudes toward PCa screening among the Jazan population. Regarding practices toward PCa, all sociodemographic data variables were nonsignificant predictors except postgraduate educational men with (*AOR* = 5.5; *p* value = 0.024] and men's family history of PCa with (AOR = 6.8; *p* value = 0.001). Further analysis showed that having a family history of PCa (AOR = 4.6, *p* = 0.001) and being married (AOR = 4.5, *p* value = 0.011) were significant predictors for higher PCa awareness. As regards attitudes toward PCa, the significant predictors that were detected were income with a *p* value (0.001, < 0.001, 0.002) and men without a family history of PCa (*p* value = 0.013), as shown in Table 6.

## 4. Discussion

This study is aimed at assessing the extent of knowledge and attitude toward PCa screening practices among men 40 and above in Jizan, Saudi Arabia. PCa is a prevalent form of cancer among men in Saudi Arabia. Recent evidence indicates that the incidence rate of PCa in Saudi Arabia is on the rise. The most affected age group comprises men over 60, accounting for 86.50% of cases [5]. The research indicated that almost half of our sample participants had poor knowledge. This value was similar and lower than some data reported from different countries. For example, in a study in Medina, Jeddah, and Makkah regions, their participants had poor knowledge (47.5%) [24]. Another study from Saudi Arabia produced similar results, where (55.2%) of participants had an inadequate level of knowledge about PCa and the PSA test [22]. In addition, two studies in Bahrain and Oman agree with our research that lack of knowledge is predominant [21, 25]. In contrast to our results, two studies from Saudi Arabia yielded a high level of knowledge about the PCa, the first from Riyadh, which showed a high level of knowledge (82.3%), while the second from Tabouk showed that the majority of study participants demonstrated good knowledge and a favourable attitude toward PCa [25]. The differences in knowledge levels among these studies may be due to variations in populations and study designs.

Our research demonstrated a significant association between knowledge level and sociodemographic variables such as marital status, educational level, residence, and monthly income. These results are consistent with Musalli et al., who showed a significant association between education and monthly income with patients' level of knowledge [23]. Socioeconomic status played an essential role in our study, in which men with a college degree or higher and those with a monthly income higher than 15,000 SR had better knowledge regarding PCa screening. Moreover, these results align with studies conducted in Italy [19] and Nigeria [27], with a significant association between education level and knowledge of PCa screening. In contrast, some research did not document a significant association between the education level and knowledge of PCa screening [14, 22]. Generally, higher educational levels are associated with better PCa screening knowledge. Education often enhances an individual's understanding of health information and resource access.

In this study, most participants showed a positive attitude toward PCa screening (60.3%). The results of two studies in Riyadh, Saudi Arabia (71%) and Italy (60%) were consistent with this finding [19, 23]. Our study's findings did not agree with those of earlier research in Saudi Arabia, Oman, Egypt, and Jordan, which revealed a negative attitude toward PCa screening [14, 24, 28]. Additionally, 85.3% of those with postgraduate degrees demonstrated the best attitude. This result differed from studies conducted in Riyadh [23], where participants older than 60 exhibited the best attitudes, and in Italy, where employed participants demonstrated the best attitudes [19]. It is common knowledge that a lack of comprehension could cause poor PSA testing outcomes.

Almost 89.5% of participants pointed out that those aged 40 should be screened for PCa and agreed that early detection is crucial. The finding was comparable to another international survey among participants across four European countries (Germany, the United Kingdom, Italy, and Spain), Canada, and the United States in late 2007, which showed that 94% think men can do the main thing to reduce their risk of PCa is to go for regular screening [15]. The study also revealed a negative attitude among the study participants toward the DRE (anal exam), perhaps because they thought it might make them more anxious and afraid, in the same context as their beliefs about the importance and effectiveness of PSA (blood test) as an essential diagnostic tool. Along the same line, a study conducted nationally in Ryiadh reflects that participants believe that PSA is an effective measure for early PCa detection (58%) [23]. The study found that about two-thirds of the male participants had no problem getting screened for PCa. However, a significant number of people still missed out on this important screening. This shows that even though they knew the screening was important, they were not motivated enough to participate in health-seeking behaviours based on the information provided.

Furthermore, we found that most survey participants had a positive attitude toward PCa screening, with about 87% agreeing that men should be screened for PCa. However, this adequate number did not seem to have significant repercussions on the practice of those patients. Thus, the level of practice among our sample was very low, with a percentage of 64% who did not visit a urologist seeking a screening for PCa. With more widespread use of PSA screening, more early-stage PCas may be detected and treated earlier [29]. Moreover, the respondents who underwent the PSA test in our sample were only 25.6%. The finding was comparable to another study conducted in South Africa, with men receiving PSA tests about 28.3% [30]. In our study, the reasons for doing the PSA test were multifactorial. However, the most crucial reason was based on advice from a doctor (25.8%), which is similar to other studies conducted globally in Italy and the United States, which found that men who had spoken with a physician about the PSA test were more likely to be screened (52.9% and 74%), respectively [19, 31]. In addition, a study conducted nationally in Ryiadh reflects that the most important reason for avoiding the PSA test is their doctors' lack of advice and guidance regarding screening and its significance (19.2%) [23]. This reflects the importance of the doctor's behaviour toward providing proper direction, which affects the patient's decision to undergo a PSA test. Such findings provide valuable information about a fundamental misunderstanding of screening concepts, which should be addressed in future local campaigns and by physicians.

### 4.1. Limitations and Strength

It is important to note that this study has some limitations. Firstly, because it followed a cross-sectional design, there were no clear associations between the independent and dependent variables. Secondly, some participants completed the questionnaire without direct assistance from the research team, which may have influenced their responses. Third, convenience sampling poses a risk of bias as it involves readily available individuals, potentially impacting the generalizability of study findings. Lastly, our study sample was limited because it was gathered through an online questionnaire, making it difficult to reach specific populations, such as elderly individuals. Despite these limitations, to our knowledge, no previous study has been conducted in the Southwest region about PCa. The results provide updated data on the knowledge and attitudes toward PCa and screening practices among men over 40 in Southwest Saudi Arabia. Therefore, the data obtained from this study could be used as a base for health education programs and for any public health intervention aimed at preventing cancer.

## 5. Conclusion

The study has revealed a significant lack of knowledge about PCa and its screening procedures among the participants. Almost 44% of the participants had poor knowledge, while 36.8% had a fair knowledge of PCa. Additionally, inadequate screening practices in the sample highlight the urgent need to provide men with comprehensive information about the advantages and disadvantages of PCa screening. Improving and expanding public health initiatives focused on raising awareness about PCa screening in the area are essential to address and rectify these knowledge gaps.

## Figures and Tables

**Table 1:** Sociodemographic characteristics of study participants in Jazan, Saudi Arabia (*N* = 468).

**Characteristics**	**N**	**%**
Age groups (years)		
40–50	265	56.6
50–59	111	23.7
60 and above	92	19.7
Nationality		
Saudi	459	98.1
Non-Saudi	9	1.9
Marital status		
Single	49	10.5
Married	378	80.8
Divorced	26	5.6
Widow	15	3.2
Educational level		
Primary	24	5.1
Intermediate	21	4.5
Secondary	82	17.5
University	246	52.6
Postgraduate	95	20.3
Residence		
Beash	54	11.5
Sabya	131	28.0
Jizan	94	20.1
Abu Areesh	84	17.9
Others governates	105	22.4
Monthly income		
Less than 5K SR	72	15.4
5K–10K SR	83	17.7
10K-15K SR	161	34.4
More than 15K SR	152	32.5
History of cancer in the family		
Do not know	121	25.9
Yes	35	7.5
No	312	66.7

Abbreviations: *N*, number; (%), percentage; SR, Saudi Riyal = $0.27.

**Table 2:** Participants' level of knowledge toward prostate cancer screening according to some selected sociodemographic characteristics (*n* = 468).

**Characteristics**	**Level of knowledge**						**p** ** value**
	**Inadequate**		**Moderate**		**Good**		
	**N**	**%**	**N**	**%**	**N**	**%**	
Age groups (years)							
40–50	113	(42.6)	101	(38.1)	51	(19.2)	0.078
50–59	41	(36.9)	45	(40.5)	25	(22.5)	
60 and above	52	(56.5)	26	(28.3)	14	(15.2)	
Nationality							
Saudi	202	(44.0)	169	(36.8)	88	(19.2)	0.965
Non-Saudi	4	(44.4)	3	(33.3)	2	(22.2)	
Marital status							
Single	24	(49.0)	17	(34.7)	8	(16.3)	0.008⁣^∗^
Married	153	(40.5)	144	(38.1)	81	(21.4)	
Divorced	17	(65.4)	8	(30.8)	1	(3.8)	
Widow	12	(80.0)	3	(20.0)	0	(.0)	
Educational level							
Primary	18	(75.0)	6	(25.0)	0	(.0)	< 0.001⁣^∗^
Intermediate	14	(66.7)	7	(33.3)	0	(.0)	
Secondary	47	(57.3)	29	(35.4)	6	(7.3)	
University	108	(43.9)	102	(41.5)	36	(14.6)	
Postgraduate	19	(20.0)	28	(29.5)	48	(50.5)	
Residence							
Beash	21	(38.9)	14	(25.9)	19	(35.2)	< 0.001
Sabya	57	(43.5)	48	(36.6)	26	(19.8)	
Jizan	35	(37.2)	36	(38.3)	23	(24.5)	
Abu Areesh	30	(35.7)	41	(48.8)	13	(15.5)	
Others	63	(60.0)	33	(31.4)	9	(8.6)	
Monthly income							
Less than 5K SR	50	(69.4)	19	(26.4)	3	(4.2)	< 0.001
5K–10KSR	37	(44.6)	30	(36.1)	16	(19.3)	
10K–15K SR	60	(37.3)	59	(36.6)	42	(26.1)	
More than 15K SR	59	(38.8)	64	(42.1)	29	(19.1)	
History of cancer in the family							
Do not know	70	(57.9)	41	(33.9)	10	(8.3)	< 0.001
Yes	11	(31.4)	12	(34.3)	12	(34.3)	
No	125	(40.1)	119	(38.1)	68	(21.8)	
The overall level of knowledge	203	(44.0)	172	(36.8)	90	(19.2)	

Abbreviations: *N*, number; (%), percentage; SR, Saudi Riyal = $0.27.

⁣^∗^*p* value based on Fisher's exact test.

**Table 3:** Participants' attitudes toward prostate cancer screening according to some selected sociodemographic characteristics (*n* = 468).

**Characteristics**	**Attitude**				**p** ** value**
	**Negative**		**Positive**		
	**N**	**%**	**N**	**%**	
Age groups (years)					
40–49	102	(38.5)	163	(61.5)	0.006
50–59	35	(31.5)	76	(68.5)	
60 and above	49	(53.3)	43	(46.7)	
Nationality					
Saudi	183	(39.9)	276	(60.1)	0.692
Non-Saudi	3	(33.3)	6	(66.7)	
Marital status					
Single	27	(55.1)	22	(44.9)	< 0.001⁣^∗^
Married	131	(34.7)	247	(65.3)	
Divorced	14	(53.8)	12	(46.2)	
Widow	14	(93.3)	1	(6.7)	
Educational level					
Primary	19	(79.2)	5	(20.8)	< 0.001
Intermediate	13	(61.9)	8	(38.1)	
Secondary	40	(48.8)	42	(51.2)	
University	100	(40.7)	146	(59.3)	
Postgraduate	14	(14.7)	81	(85.3)	
Residence					
Beash	20	(37.0)	34	(63.0)	0.254
Sabya	49	(37.4)	82	(62.6)	
Jizan	38	(40.4)	56	(59.6)	
Abu Areesh	28	(33.3)	56	(66.7)	
Others	51	(48.6)	54	(51.4)	
Monthly income					
Less than 5K SR	56	(77.8)	16	(22.2)	< 0.001
5K–10K SR	31	(37.3)	52	(62.7)	
10K–15 K SR	49	(30.4)	112	(69.6)	
More than 15K SR	50	(32.9)	102	(67.1)	
History of cancer in the family					
Do not know	73	(60.3)	48	(39.7)	< 0.001
Yes	13	(37.1)	22	(62.9)	
No	100	(32.1)	212	(67.9)	
General Attitude	186	(39.7)	182	(60.3)	

Abbreviations: *N*, number; (%), percentage; SR, Saudi Riyal = $0.27.

⁣^∗^*p* value based on Fisher exact test.

**Table 4:** Participants' attitudes toward prostate cancer screening (*n* = 468).

**Statement**	**N**	**%**	**Mean (SD)**
Above 40 years old should do a screen			
Strongly agree	240	51.3%	1.6 (0.8)
Agree	167	35.7%	
Not sure	50	10.7%	
Disagree	9	1.9%	
Strongly disagree	2	0.4%	
Early detection protects from serious effects			
Strongly agree	281	60.0%	1.5 (0.8)
Agree	138	29.5%	
Not sure	39	8.3%	
Disagree	7	1.5%	
Strongly disagree	3	0.6%	
An anal exam is not acceptable			
Strongly agree	119	25.4%	2.6 (1.2)
Agree	100	21.4%	
Not sure	140	29.9%	
Disagree	86	18.4%	
Strongly disagree	23	4.9%	
Blood tests are not final			
Strongly agree	28	6.0%	3.5 (1.1)
Agree	40	8.5%	
Not sure	166	35.5%	
Disagree	158	33.8%	
Strongly disagree	76	16.2%	
Blood tests are expensive			
Strongly agree	59	12.6%	2.8 (1.0)
Agree	96	20.5%	
Not sure	229	48.9%	
Disagree	69	14.7%	
Strongly disagree	15	3.2%	
Essential to do the anal exam			
Strongly agree	37	7.9%	3.0 (1.1)
Agree	122	26.1%	
Not sure	181	38.7%	
Disagree	77	16.5%	
Strongly disagree	51	10.9%	
The anal exam is embarrassing			
Strongly agree	161	34.4%	2.4 (1.3)
Agree	115	24.6%	
Not sure	79	16.9%	
Disagree	93	19.9%	
Strongly disagree	20	4.3%	
Regular screening leads to early detection			
Strongly agree	177	37.8%	3.5 (1.1)
Agree	174	37.2%	
Not sure	104	22.2%	
Disagree	8	1.7%	
Strongly disagree	5	1.1%	
Cancer tests increase my anxiety			
Strongly agree	32	6.8%	2.3 (0.9)
Agree	54	11.5%	
Not sure	110	23.5%	
Disagree	182	38.9%	
Strongly disagree	90	19.2%	
Even if busy, will do tests			
Strongly agree	99	21.2%	1.6 (0.5)
Agree	187	40.0%	
Not sure	149	31.8%	
Disagree	24	5.1%	
Strongly disagree	9	1.9%	

Abbreviations: *N*, number; (%), percentage; SD, standard deviation.

**Table 5:** Participants' practices regarding prostate cancer screening according to age groups (*n* = 468).

**Statement**	**All participants**		**Age groups (years)**						**p** ** value**
			**40–49**		**50–59**		**60 and above**		
	**N**	**%**	**N**	**%**	**N**	**%**		**%**	
Visited a urologist for screening									
Yes	167	(35.7)	73	(27.5)	47	(42.3)	47	(51.1)	< 0.001
No	301	(64.3)	192	(72.5)	64	(57.7)	45	(48.9)	
The urologist requested PSA									
Yes	138	(29.5)	65	(24.5)	39	(35.1)	34	(37.0)	0.026
No	330	(70.5)	200	(75.5)	72	(64.9)	58	(63.0)	
I did PSA									
Yes	120	(25.6)	57	(21.5)	34	(30.6)	29	(31.5)	0.064
No	348	(74.4)	208	(78.5)	77	(69.4)	63	(68.5)	
Why did you do the PSA test?									
Advice from Doctor	47	(25.8)	27	(24.3)	11	(26.8)	9	(30.0)	0.004
I am at high risk	23	(12.6)	7	(6.3)	10	(24.4)	6	(20.0)	
Participation in the Wegaya program	42	(23.1)	29	(26.1)	7	(17.1)	6	(20.0)	
To detect cancer early	28	(15.4)	13	(11.7)	8	(19.5)	7	(23.3)	
Other	42	(23.1)	35	(31.5)	5	(12.2)	2	(6.7)	
When are you did PSA?									
less than 30 years	29	(16.6)	27	(24.3)	2	(5.6)	0	(.0)	< 0.001⁣^∗^
30–40	27	(15.4)	24	(21.6)	1	(2.8)	2	(7.1)	
40–50	65	(37.1)	47	(42.3)	11	(30.6)	7	(25.0)	
50–60	43	(24.6)	11	(9.9)	21	(58.3)	11	(39.3)	
Above 60	11	(6.3)	2	(1.8)	1	(2.8)	8	(28.6)	
Why did you not do PSA?									
Not advised by the doctor	11	(3.5)	7	(3.8)	3	(4.1)	1	(1.6)	0.065
I am not at high risk	130	(40.9)	76	(41.5)	35	(47.3)	19	(31.1)	
Time limitation	51	(16.0)	29	(15.8)	8	(10.8)	14	(23.0)	
Afraid of cancer detection	18	(5.7)	6	(3.3)	5	(6.8)	7	(11.5)	
Others	70	(22.0)	46	(25.1)	10	(13.5)	14	(23.0)	
Anxious	38	(11.9)	19	(10.4)	13	(17.6)	6	(9.8)	
If not, will you do it in the future?									
Yes	332	(70.9)	186	(70.2)	84	(75.7)	62	(67.4)	0.398
No	136	(29.1)	79	(29.8)	27	(24.3)	30	(32.6)	

Abbreviations: *N*, Number; (%), percentage; SR, Saudi Riyal = $0.27.

⁣^∗^*p* value based on Fisher's exact test.

**Table 6:** Multivariate logistic regression analysis for the factors associated with practices, knowledge, and attitudes toward prostate cancer screening among the Jazan population.

**Category**	**Subcategory**	**Practices**				**Knowledge**				**Attitudes**			
		**p**	**aOR**	**95% CI for aOR**		**p**	**aOR**	**95% CI for aOR**		**p**	**aOR**	**95% CI for aOR**	
				**Lower**	**Upper**			**Lower**	**Upper**			**Lower**	**Upper**
Marital status	Single (REF)		1				1				1		
	Married	0.737	1.1	0.6	2.3	0.011	4.5	1.4	14.3	0.282	1.4	0.7	2.8
	Divorced	0.528	0.7	0.2	2.2	0.997	1.0	0.2	5.7	0.672	1.3	0.4	3.7
	Widow	0.604	0.6	0.1	3.8	0.542	1.8	0.3	11.9	0.118	0.2	0.0	1.6

Education	Primary (REF)		1				1				1		
	Intermediate	0.212	0.3	0.0	2.1	0.599	0.7	0.1	3.1	0.643	0.7	0.1	3.3
	Secondary	0.984	1.0	0.2	3.9	0.590	0.7	0.2	2.5	0.873	1.1	0.3	4.0
	University	0.520	1.6	0.4	6.6	0.455	0.6	0.2	2.3	0.748	0.8	0.2	3.1
	Postgraduate	0.024	5.5	1.3	24.2	0.211	2.5	0.6	10.0	0.176	2.7	0.6	11.3

Income	Less than 5K SR (REF)		1				1				1		
	5K–10K SR	0.551	1.3	0.5	3.2	0.381	1.5	0.6	3.7	0.001	4.0	1.8	9.0
	10K–15K SR	0.165	1.8	0.8	4.4	0.720	1.2	0.5	3.1	< 0.001	4.6	2.0	10.5
	More than 15K SR	0.570	1.3	0.5	3.1	0.556	0.7	0.3	2.0	0.002	3.8	1.6	8.8

History of prostate cancer	Do not know (REF)		1				1						
	Yes	0.001	6.8	2.7	17.2	0.001	4.6	1.9	10.9	0.178	1.8	0.8	4.2
	No	0.060	1.7	1.0	2.9	0.166	0.7	0.4	1.2	0.013	1.9	1.1	3.2

Age groups	40–50 years (REF)		1				1				1		
	50–59 years	0.122	1.5	0.9	2.5	0.162	1.5	0.9	2.6	0.372	1.3	0.8	2.1
	60 and above years	0.899	1.0	0.5	1.7	0.048	1.9	1.0	3.6	0.636	0.9	0.5	1.5

Abbreviations: aOR, adjusted odds ratio; CI, confidence interval; *p*, *p* value.

## Data Availability

The data presented in this study are available on request from the corresponding author.
